# Intravascular Lithotripsy: Approach to Advanced Calcified Coronary Artery Lesions, Current Understanding, and What Could Possibly Be Studied Next

**DOI:** 10.3390/jcm13164907

**Published:** 2024-08-20

**Authors:** Giorgi Kochiashvili, Natalia Fongrat, Bhavana Baraskar, Biruk Amare, Micaela Iantorno

**Affiliations:** 1 Internal Medicine, Mary Washington Healthcare, Fredericksburg, VA 22401, USA; natalia.fongrat@mwhc.com (N.F.); bhavana.baraskar@mwhc.com (B.B.); biruk.amare@mwhc.com (B.A.); 2 Oracle Heart & Vascular, Fredericksburg, VA 22401, USA; micaela.iantorno@mwhc.com

**Keywords:** intravascular lithotripsy, calcification, coronary artery calcification, coronary artery disease, atherectomy, percutaneous coronary intervention

## Abstract

Calcified and resistant narrowing of arteries poses significant difficulty in performing percutaneous coronary interventions (PCIs), as they increase the risk of subpar outcomes leading to worse clinical outcomes. Despite the existence of dedicated technologies and devices, including various balloons and atherectomy systems, they often do not ensure sufficient plaque modification and ideal vessel preparation for optimal stent deployment. Intravascular lithotripsy (IVL), a technology originally developed for urological procedures, has recently been used to safely and selectively disrupt calcified depositions in both peripheral and coronary arteries by sonic waves that seamlessly transfer to nearby tissue, enhancing vessel compliance with minimal impact on soft tissues. In the coronary arteries, the use of IVL plays a role in the process of “vessel preparation” before the placement of stents, which is crucial for restoring blood flow in patients with severe coronary artery disease (CAD), and is considered a minimally invasive technique, reducing the need for open heart surgeries and associated risks and complications. Studies have shown that IVL can lead to improved procedural success rates and favorable long-term outcomes for patients with severely calcified coronary artery disease. With the advent of IVL, the disruption of severe calcification of coronary artery and stenotic lesions before stent implantations can be performed. Despite promising data for treating calcified lesions, IVL is significantly underutilized in clinical practice, long-term clinical data and extensive research are needed to validate its further safety and efficacy. In this article, we reviewed the literature discussing the use of IVL in the coronary arteries as an approach for addressing intravascular atherosclerotic plaques, particularly focusing on heavily calcified plaques that are resistant to standard initial PCI, while also evaluating its safety in comparison to alternative methods.

## 1. Introduction

Vascular calcification refers to the accumulation of calcium primarily in the extracellular space within the inner layers of arteries known as the intima and media; less frequently, deposition may also occur in the intracellular space, though its significance remains unclear [1,2]. Studies indicate a significant correlation between coronary artery calcification and the subsequent development of atherosclerosis. A valuable method for evaluating this is through the coronary artery calcium score and the Framingham Risk Score (FRS), which utilizes a range of diverse risk factors and comorbidities to forecast the likelihood of nonfatal myocardial infarction (MI) or mortality linked with CAD [3,4]. It is crucial to intervene in severely diseased coronary arteries to prevent adverse cardiac outcomes. Severely calcified arteries present challenges for intervention, often requiring preparation before PCI. IVL offers a novel approach to preparing these arteries, making them more amenable to PCI [5]. Other modalities for vessel preparation that are being used include the use of standard or high-pressure balloons, cutting or scoring balloons, and different atherectomy devices such as orbital atherectomy (OA) and rotational atherectomy (RA). Atherectomy may be associated with a high risk of complications including vessel dissection, occlusion, embolization, or perforation [6]. IVL could represent a more effective alternative approach compared to atherectomy [7].

## 2. Description of Intravascular Lithotripsy and an Overview of Its Use, Safety, and Success Rate

In cases where traditional methods like high-pressure dilation for stent placement are ineffective in preparing calcified coronary arteries, IVL emerges as a viable alternative. This technique employs low-pressure pulsatile sonic waves to disrupt calcified lesions [8]. 

Shockwave pulses effectively target calcium deposits regardless of their depth within the vessel wall, unlike RA or OA, which struggle to modify deeply seated calcium [9,10]. 

In a cohort study, IVL demonstrated success in reducing in-stent stenosis in 61 out of 78 lesions (78.2%). Notably, IVL proved to be beneficial particularly for patients who experienced stent stenosis with ineffective high-pressure dilation, expanding the stents and resulting in reduced in-stent stenosis in 64.7% of occurrences [5]. 

Interventional cardiologists can perform IVL without extensive additional training, as it is compatible with all coronary guidewires in comparison with the other approaches, such as atherectomy, which needs further qualification. To begin, the shockwave balloon must be appropriately sized based on the reference vessel diameter, maintaining a 1:1 ratio. It should then be positioned within the target calcified lesion and inflated up to 2–4 atm pressure to ensure proper contact with the vessel wall, delivering acoustic pulses in cycles of 10. Subsequently, the balloon can be inflated further up to 6 atm (nominal pressure) to enhance compliance and confirm symmetrical expansion, indicating successful calcium modification. Careful deflation of the balloon is necessary to release any small air bubbles. These steps should be repeated for each intended IVL cycle, with a recommendation of at least two cycles for effective treatment of the target area. The repositioning of the catheter may be required for lesions longer than 12 mm, potentially leading to overlapping treatment areas. In certain situations, due to the slightly larger size of the shockwave catheter, preliminary dilatation with standard balloons may be necessary to aid in its delivery and positioning, especially when there is significant narrowing of the lumen. However, the catheter allows for the utilization of guide-catheter extenders and buddy-wire support. Additionally, it is labeled as compatible with six French catheters but can also be used with five French guiding catheters in cases where the radial artery is narrower. Furthermore, although not obligatory, post-IVL dilatation with noncompliant balloons could be considered to further widen the lumen. Moreover, in challenging lesions, adjunctive therapies such as cutting/scoring balloons or atherectomy devices may be employed to enhance outcomes. IVL seems beneficial for modifying coronary calcium to enhance stent expansion. It eliminates the necessity for intricate lesion preparation methods like RA, except for severe cases where IVL is not feasible [11,12].

The Disrupt CAD I study was conducted on 60 patients between December 2015 and September 2016 at seven hospitals in Europe and Australia. These patients had severe calcifications of coronary arteries with a median diameter stenosis of 72.5% (range 58.5–77.0) and a median lesion length of 18.2 mm. The primary outcome of the study was the reduction in stenosis with a residual diameter of <50% without in-hospital major adverse cardiovascular events (MACEs). The results indicated a significant success rate of stent delivery in 100% of cases, reducing the stenosis to a mean of 12.2% (range 6.7–20.5%) with a success rate of 95% in a total of 57 patients. IVL was associated with three (5%) periprocedural non-Q wave MIs that qualified as MACE. Notably, in this study, no instances of perforation, embolization, or vessel occlusion were observed [8].

## 3. Comparison of Intravascular Lithotripsy to Some Other Available Approaches 

In recent years, there has been increasing attention paid to comparing the effectiveness, safety, and overall clinical results of balloon angioplasty, atherectomy, and IVL in treating CAD and peripheral artery disease (PAD) [13]. Balloon angioplasty is a commonly employed method for treating occlusive disease across various arterial segments. However, the current outcomes are not entirely satisfactory in severely calcified lesions. Some patients may experience under-expanded balloon/stenting or balloon rupture during angioplasty. Stent under-expansion is associated with a higher incidence of restenosis or re-occlusion [14].

Atherectomy entails mechanically altering and eliminating plaque by utilizing different tools like rotational, orbital, or laser-based systems. It is a minimally invasive procedure that involves the removal of atherosclerotic plaque from blood vessels using various mechanical devices. The goal of atherectomy is to debulk or modify the plaque, restoring blood flow and improving vessel patency. In RA, a high-speed rotating burr abrades and pulverizes calcified plaque [15]. In OA, a diamond-coated crown rotates eccentrically within the vessel, sanding away plaque while minimizing damage to healthy vessel walls [16]. Laser atherectomy can be particularly beneficial in lesions that are difficult to treat with other techniques, such as heavily calcified or fibrotic lesions [17]. Finally, directional atherectomy (DA) involves the use of a cutting blade or a small rotating cutter to remove plaque from the vessel wall. This technique allows for precise plaque removal and is often used in conjunction with balloon angioplasty and stent placement [18,19]. 

A retrospective analysis carried out at a single medical center compared patients who underwent percutaneous coronary interventions using RA or IVL for heavily calcified coronary lesions. The study involved 25 patients, with 13 in the RA group and 12 in the IVL group. The results indicated that the IVL group exhibited significantly larger final minimal lumen areas (7.6 mm^2^ vs. 5.4 mm^2^; *p* = 0.01), greater lumen area gain (4.1 mm^2^ vs. 2.3 mm^2^; *p* = 0.01), and higher final stent volumes (491.2 mm^3^ vs. 326.2 mm^3^; *p* = 0.03) [7]. 

A cohort study aimed to evaluate and compare in-stent pressure gradients, assessed by vessel fractional flow reserve (vFFR), in calcified lesions treated with either RA or IVL. The study involved patients undergoing PCI with RA or IVL from two European centers. The primary outcome was the measurement of post-PCI in-stent vFFRgrad. Secondary outcomes included determining the percentage of patients achieving complete functional revascularization, defined as distal vFFR post-PCI (vFFRpost) ≥ 0.90. Among the initially screened 210 patients, 105 matched patients (70 in the RA group and 35 in the IVL group) were included. Following PCI, the IVL group showed notably lower in-stent pressure gradients compared to the RA group (0.032 ± 0.026 vs. 0.043 ± 0.026, respectively; *p* = 0.024). However, the proportions of vessels achieving complete functional revascularization were similar between the RA and IVL groups (32.9% vs. 37.1%, respectively; *p* = 0.669) [20].

A retrospective study examined data from a registry established in two collaborative, high-volume cardiac centers specializing in PCI: the Department of Cardiology at The Copper Health Centre (MCZ) in Lubin, Poland, and the Department of Cardiology at the Provincial Specialized Hospital in Legnica, Poland. The study population included individuals from all consecutive patients with calcified lesions who underwent PCI and necessitated further lesion preparation using either RA or IVL for left main artery (LM) diseases (a primary inclusion criterion for the study). During the study period, 45 consecutive patients who underwent PCI for LM lesions and were assisted by either RA or IVL were enrolled. In the RA cohort, three fatalities were recorded that transpired during hospitalization. One occurred periprocedurally, involving a patient who suffered cardiac arrest before the procedure and underwent the intervention with assistance from a Lucas CPR assist device. Subsequently, two deaths ensued post-procedure; these patients were transferred to a local intensive care unit (ICU) due to multiorgan dysfunction following PCI, and their deaths were reported 10 and 17 days after the procedure. A noteworthy issue is an additional death that occurred 17 days after discharge, involving a patient with multiple comorbidities and a history of alcohol abuse. Moreover, one instance of MI affecting a previously untreated vessel was noted in the RA group 120 days post-procedure. In the subgroup treated with IVL, one case of fatal in-stent thrombosis occurred during the hospital stay (five days post-PCI). Additionally, following discharge (14 days post-procedure), a second death was observed in the IVL cohort. The deceased had multiple comorbidities and a low left ventricular ejection fraction (LVEF) of 25% and was under clinical observation before the scheduled implantation of an implantable cardioverter-defibrillator (ICD) [21]. 

Primary balloon angioplasty remains a viable initial strategy for an ST-Segment Elevation Myocardial Infarction (STEMI) caused by a heavily calcified stenosis that cannot be dilated, as long as TIMI 3 flow is attainable. In certain clinical situations, RA and IVL can complement each other. When facing calcified lesions that are challenging for balloon crossing, RA is the preferred method to facilitate the advancement of balloons and stents. If the lesion remains resistant to dilation after RA, a hybrid approach incorporating additional intracoronary lithotripsy can be an effective strategy, further modifying the calcified plaque and facilitating stent delivery [22].

Combined utilization of OA followed by IVL was examined in a case involving an 81-year-old male who presented with chest pain and was subsequently diagnosed with non-ST-Segment Elevation Myocardial Infarction (NSTEMI). As coronary angiography revealed triple vessel disease, and due to chronic obstructive pulmonary disease (COPD) and moderate-to-severe aortic stenosis, the patient was deemed a high-risk candidate for Coronary Artery Bypass Grafting (CABG). Initial attempts to address heavily calcified lesions in the right coronary artery (RCA), with an area of 1.55 mm^2^, involved IVL, which did not result in significant calcium cracking after eight cycles due to the severity of calcification. Subsequently, a decision was made to proceed with high-risk OA for the mid-RCA, successfully debulking the calcium and creating a characteristic “snowman” appearance with an increased luminal gain of 2.23 mm^2^. However, despite this improvement, stent placement was still deemed inadequate, prompting the use of IVL, which yielded excellent outcomes in terms of luminal area. Ultimately, two drug-eluting stents were successfully deployed overlappingly [23]. Another case report demonstrates another successful application of the combination of OA and IVL in the Left Anterior Descending artery (LAD), resulting in excellent stent expansion [24]. Although larger trials are needed to understand the safety and efficacy of combining OA and IVL, these two case reports demonstrated favorable outcomes without complications.

## 4. Use of Intravascular Lithotripsy in ST-Segment Elevation Myocardial Infarction Patients

Although evidence supporting IVL with PCI for STEMI is lacking, there have been reports of a few cases where IVL was successfully used for vessel preparation for stenting in STEMI patients. Performing PCI in STEMI patients can be challenging due to calcified coronary plaques. These cases represent the initial documentation of IVL application in primary PCI for STEMI. A case series with three cases documented promising outcomes from using IVL in STEMI patients, with all three achieving minimal residual stenosis post-IVL preparation of the coronary followed by stent placement. Although there was one occasion of ventricular tachycardia that required six shocks, the final angiographic result was favorable. Despite limited data being available on the use of IVL in PCI for STEMI, early experiences showing favorable outcomes and no reported complications in these cases are promising [25].

## 5. Risks and Complications Associated with Intravascular Lithotripsy

While IVL is generally considered safe and effective, like any medical procedure, it carries certain risks and potential complications. Several studies have been conducted to analyze the potential complications of IVL. Some of the complications associated with IVL found were coronary artery perforation and dissection, vascular injuries such as hematoma formation and arrhythmias, and the embolization of calcified debris, resulting in stroke or transient ischemic attack (TIA). Recently, in 2023, data were analyzed from the FDA MAUDE database for adverse events associated with coronary IVL. They reported incidence of catheter dislodgement in 26% of cases, coronary artery dissection and balloon rupture in 23%, death in 17%, around a 14% risk of arrhythmias, perforation in 8% of cases and thrombus formation and balloon kinking in nearly 5% of cases [26]. Notably, before the FDA MAUDE database analysis, the Disrupt Coronary Artery Disease (DISRUPT CAD I) trial in 2021 with 628 patients showed coronary perforation in 0.2% of cases after stent placement, coronary dissection post IVL in 1.8% of cases, and death occurring in 0.2% of cases [27]. Later on, a study [28] comparing success rates and complications of IVL between general and tertiary hospital settings reported four incidences of ruptured IVL balloons in tertiary hospital settings, whereas in general hospital settings, it noted one incidence each of coronary dissection and type 5 distal coronary perforation. In 2022, Sattar et al. also performed a meta-analysis to study the safety and efficacy of IVL. In their analysis, the leading complications after 1 month of follow-up observed were MI (6.5%) and target vessel revascularization (1%). Coronary dissection was observed in 1.7% of cases among in-hospital adverse events [29]. Of note, a case review of the use of IVL in non-coronary artery lesions reported outcomes including the occurrence of complications in 12% of the total 60 cases; among those, dissections contributed to 4.7%, while vessel perforation, transient hypotension and TIA accounted for 1.6% each. It is important to note that the likelihood of these complications occurring varies depending on factors such as the severity of the calcification and the skill and experience of the healthcare team performing the procedure. The percentages of these complications are negligible when compared to the advantages of IVL [30].

An example of a perforation of the LAD following the discovery of chronic total occlusion occurred in a 68-year-old female. The patient initially presented with angina and shortness of breath, subsequently being diagnosed with significant anterior ischemic defects on myocardial scintigraphy. The initial decision was made to perform PCI by preparing the patient with the use of a 4.0 mm noncompliant balloon, which resulted in unsuccessful stent placement. Subsequently, IVL was employed, delivering 40 intracoronary shockwaves. Although this resulted in favorable stent expansion, it led to coronary artery perforation behind the stent struts. Initial measures, including the sealing of the shockwave balloon and intravenous protamine, were not sufficient to stop the leak. Traditional methods of treating coronary perforation, using a covered stent, were employed, successfully stopping the leak [31].

## 6. Limitations of Intravascular Lithotripsy

In addition to the above-mentioned complications, IVL possesses certain limitations. According to Ali et al., IVL was found to be less effective in heavily calcified and complex lesions with significant eccentric calcium deposits and nodular calcium, potentially leading to incomplete lesion preparation [10]. Typically, due to incomplete preparation, the efficacy of IVL decreases and the probability of complications rises [32]. In some cases, IVL may not even completely modify or fracture the calcium, giving rise to additional interventions such as atherectomy or scoring balloon angioplasty to achieve optimal lesion preparation [32,33]. Though a formal qualification as an interventional cardiologist is not required to perform the procedure, the successful use of IVL requires training and expertise. Inexperienced operators may face challenges in optimizing device positioning and energy delivery, potentially affecting procedural outcomes [33,34]. Moreover, IVL requires specialized equipment, which can be costly and may not be universally available in all healthcare settings, potentially limiting its widespread adoption [35]. Despite promising results of IVL, some studies suggest that there are still a lack of substantial clinical data that prove the long-term efficacy and safety of IVL compared to alternative treatment modalities such as atherectomy. Overall, while IVL represents a valuable tool in the treatment of calcified coronary lesions, its limitations should also be considered and should be weighed against potential benefits on a case-by-case basis [8].

## 7. Conclusions

IVL stands as a beacon of innovation in the realm of interventional cardiology, offering a promising alternative to traditional methods for managing calcified CAD and PAD. Its efficacy in fragmenting calcium deposits through pulsatile sonic waves ensures safer and more effective vessel preparation for stent placement, thus enhancing luminal gain without extensive training requirements compared to atherectomy techniques. The unique ability of IVL to modify deeply embedded calcium, unreachable by conventional atherectomy, positions it as a pivotal tool in the cardiologist’s arsenal, especially in cases where high-pressure dilation fails.

Despite its success, IVL is not without its limitations and potential complications, such as coronary artery perforation, dissection, and the risk of arrhythmias, among others. However, the relatively low incidences of these adverse events, when juxtaposed with the high success rates of stent delivery and a significant reduction in stenosis, underscores the safety and effectiveness of IVL as a therapeutic modality. Furthermore, the advent of combining IVL with other techniques, such as RA or OA, in challenging lesions has demonstrated enhanced outcomes, suggesting a synergistic potential that warrants further exploration.

The evolution of IVL from a novel approach to a validated treatment underscores the importance of continued research and clinical trials to fully elucidate its capabilities and limitations. As we advance, it is crucial to balance enthusiasm for innovative technologies with rigorous assessment of their long-term efficacy and safety. IVL, with its proven utility in modifying calcified lesions and facilitating stent expansion, undoubtedly marks a significant advancement in the treatment of CAD and PAD. Yet, the quest for perfection in medical interventions continues, as does our commitment to refining these technologies for the betterment of patient care.
